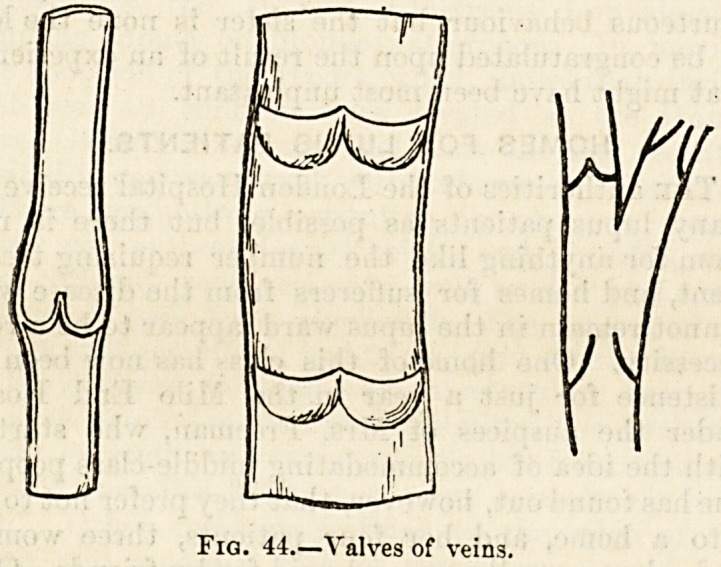# The Hospital. Nursing Section

**Published:** 1902-05-31

**Authors:** 


### The Hospital.

### Nursing Section.

Contributions for this Section of "The Hospital" should be addressed to the Editor, "The Hospital" Nursing Section, 28 & 29 Southampton Street, Strand, London, W.C.

No. 818.—Vol. XXXII. *SATURDAY, MAY* 31, 1902.

### Notes on News from the Nursing World.

### OUR CORONATION HOSPITAL SUNDAY NUMBER.

A double number of The Hospital will be issued on Saturday, June 14th, the price of which will be 4d. instead of 2d., as usual. This number will contain two excellent photogravure plates, being portraits of the Prince and Princess of Wales, produced from new portraits of their Royal Highnesses, with autographic signatures. The plates are suitably mounted for framing. Subscribers desiring to obtain these plates must send two penny stamps, before June 11th, to the Manager of The Hospital, 28 Southampton Street, W.C., as they will not otherwise be issued with subscription copies.

### ARMLETS FOR CORONATION WEEK.

An excellent suggestion has been made by some of the members of the Royal National Pension Fund for Nurses. It is proposed that the armlet should be worn during Coronation week. Of course, it can be worn whenever a member chooses, but it will be particularly in accordance with the fitness of things if every Pension Fund nurse wears her decoration daily from June 15th to the end of the month.

### LIVERPOOL NURSES AND THE CORONATION FESTIVITIES.

The Lord Mayor of Liverpool, who has always shown sympathy with the nursing movement, has arranged to afford the hospital nurses of Liverpool the opportunity of witnessing from the deck of a steamer the Mersey display during the Coronation festivities. As may be imagined, the hospital nurses in the city are greatly delighted at the thoughtfulness of the chief magistrate, who has thus assured them a good time without expense or trouble.

### WAITING ON THE KING'S GUESTS.

A nurse living in Bayswater, who was very much taken with the idea of nurses waiting on the King's guests at the dinner to be given to the poor of London on July 5th, acted upon the advice given in The Hospital of May 10th and wrote to the Lord Mayor offering the services of her sister and herself, with the result that the next morning she received a letter thanking her for the offer, which was gladly accepted. During the evening of the same day she had a visit from an official who said that he had 258 stewards to find, and so far only knew of three, and would be glad to hear of any ladies, nurses or otherwise, who would volunteer their services. On the other hand, a nurse at Southampton who sent in her name some days since to the Lord Mayor has had no reply.

### THE WAR NURSES.

Nursing Sisters L. L. Langley Naylor, M. Smyth, A. M. Winder, and L. L. Watts, A.N.S.R., arrived at Southampton on board the *Simla* on the 22nd inst. and rejoin the ship. Civil Nurse H. Kenealy also rejoins. F. Donald, A.N.S.R., requires one month's leave, and returns to South Africa. A. E. Turner, A.N.S.R., was invalided home and granted three months' leave. On board the *Orient*, arriving the same day, were L. M. Monk, A.N.S.R., no leave required; A. Lee-Smith, A.N.S.R., two months; E. Hoadley, New South Wales A.N.S.R., six weeks; K. M. Champion, Civil Nurse, two months; E. B. Norrie, Civil Nurse, six months. All return to South Africa. A. K. Statham, A.N.S.R., was invalid home and granted three months' leave.

### A NEW BRANCH AT HASLAR.

It is probable that one or two additions to the nursing staff at the Royal Naval Hospital, Haslar, will be required shortly. The large building which has been erected for the reception of patients suffering from infectious diseases is now completed. It is in four blocks, and there are a hundred beds. The nursing will, of course, be done by the sisters, and it is obvious from the details we published last week that the existing staff is not large enough to undertake fresh duties.

### THE NURSING AT BELLEVUE HOSPITAL, NEW YORK.

The rumour that the nursing of male patients at Bellevue Hospital, New York, is, in the near future, to be done by women instead of by men, has obtained currency on this side of the Atlantic. We are officially informed that a change in the nursing system "is contemplated," but that at present no definite information can be given. The precise nature of the proposed change is a legitimate subject for curiosity, but there is little doubt that it lies in the direction of the extension of the number of female nurses.

### A NEW FLOOR AT THE LONDON HOSPITAL.

In connection with other developments at the London Hospital, a new floor for nurses' rooms has been added. This provides about 20 bedrooms, and sisters' rooms, running along a corridor, and at each end are bath-rooms and lavatories. A conveniently arranged gas stove has also been fixed, where water can be boiled at short notice for tea-making, etc. The rooms, which have an extensive view over the chimney-pots of East London, are very simply furnished; the walls are coloured terra-cotta. A wide staircase, or one of the new electric lifts, is the mode of approach to this floor, which is immediately above some of the rooms of the new operating department.

### DAMAGES £600.

We heartily congratulate Miss Rosalie Mansell upon the result of her action for libel against the *Sun* newspaper. Miss Mansell, last year, became superintendent nurse at the Renfrew Road Workhouse Infirmary, Lambeth, and her case was that scandalous and untrue accusations were made against her by an inmate of the workhouse at a meeting of the Board of Guardians, and were published in the

*Sun.* Miss Mansell indignantly denied these allegations, but her denial was ignored, a point which Mr. Justice Grantham strongly emphasised in his summing up. The jury found a verdict for the plaintiff, and awarded her £600—exemplary damages, but none too heavy in the circumstances.

### SOMERSET HOSPITAL, CAPETOWN.

AN the annual meeting of the board of management of the New Somerset Hospital, Capetown, it was stated that a further sum of £5,000 was required to complete the nurses' home and the surgeon's residence. The removal of the nurses' quarters from the hospital will allow of two additional wards being opened. Great regret was expressed at the resignation of the matron, sister Mary Agatha, of the All Saints' Community, and it was agreed to present her with a sum of money which she might like to use for charitable purposes. Mention was made of the heroism displayed by the Misses Keysel, the two ladies who died whilst they were engaged in nursing plague patients, and a suggestion was made that a bed should be endowed as a memorial of these noble women.

### INVALID CHILDREN'S HOME.

DR. ELIZABETH GARRETT ANDERSON presided at the annual meeting in University Hall, Gordon Square, of the Invalid Children's Convalescent Nursing Home on Monday afternoon. In moving the adoption of the report, which shows that during last year 48 children were treated at the Home, Mrs. Garrett Anderson said that she had recently visited it and was struck with its home-like character. "There was nothing institution-like about it; everything was extraordinarily bright and cheerful, and the children were certainly well tended and their sufferings had been alleviated. She was glad to notice that something had to be paid by the friends of the patients, for that was calculated to keep the parents in mind of their responsibility. The smallness of the expenditure was remarkable, but from what she herself saw the little ones were exceedingly well fed." Other speakers testified to the value of the work, and a cordial vote of thanks was accorded to the medical officers and the lady superintendent of the home.

### QUEEN'S NURSES AT CHATHAM.

A FEATURE of the fifth annual report of the Chatham Nursing Association, which was adopted at the meeting, was the statement that a preponderating number of housewives had been nursed. One of the advantages which people derive from the establishment of district nursing is that when a wife is taken ill she can be nursed in her own home so long as the case is not of an infectious character. It is not satisfactory that 262 visits were paid in the year to cases which, "on investigation, proved unsuitable for nursing." Why, and by whom, were the nurses sent to such cases? We observe that of the 341 new cases nursed, 272 were sent by medical men, 12 by district visitors, 44 by patients' friends, 10 being found by the nurses. The district visitors and patients' friends are not, as a rule, qualified to judge whether the services of a nurse is required. This should, if possible, be left for a medical man to determine. It is interesting to learn that Lord Raglan, the Under Secretary of State for War, sent the Association a touching letter of sympathy. There is reason to hope that the nurses' home may not only be commenced but finished this year. At present, the nurses have comfortable, but inadequate, quarters, and it will be a great boon to them to be installed in the Queen Victoria Home. The income exceeded the expenditure by nearly £30. Altogether, the prospects of the Association seem to be excellent.

### MATRON AND SUPERINTENDENT NURSE.

AN interesting discussion took place at the recent meeting of the Belfast Board of Guardians. It was proposed by a Committee of the Board that the superintendent nurse at the Infirmary should be "promoted" to a new position and be called the matron of the nurses' home, and that a new superintendent nurse should be appointed. The chairman, upon being challenged as to whether they had the power to transfer the superintendent to the post of matron of the nurses' home, stated that the scheme had the approval of the Inspector of the Local Government Board; and though it was ultimately decided not to accept the recommendation of the Committee, we cannot see any reason why, if the superintendent nurse wishes to become matron of the nurses' home, she should not be appointed to that office.

### SOUTH LONDON DISTRICT NURSING ASSOCIATION.

THE eighteenth annual meeting of the South London District Nursing Association was held at Clapham Rectory under the presidency of Canon Erskine Clarke, chairman, who himself, in moving the adoption of the report, said that "the nurses were a pattern to them all in the diligence with which they pursued their work and the enormous amount of energy they devoted to the care of the poor." The report showed that there has been a great development in the work of the Association, and that last year 1,549 cases were nursed and 28,973 visits paid. In addition to these, a nurse, who was specially appointed to visit the schools and look after the minor ailments of the children, made upwards of 900 attendances. Unfortunately, the expenditure exceeded the income by £21, a sufficient incentive for the supporters of the organisation to increase rather than to relax their efforts on its behalf. The Bishop of Rochester observed that he was there that day to express his sympathy with the work of the Association and to hear a little about it, and not from any thought that he would be able to instruct those present about it; still, at a time when perhaps the divisions between Christian bodies were rather accentuated, it was a great pleasure to feel that in this matter they could co-operate with hearty accord. Dr. Annie McCall spoke of the value of the Association in raising the *status* of the nurses, and Miss Honnor Morten referred especially to the work of the nurse appointed to visit the elementary schools.

### CASHING THE CHEQUES OF INFIRMARY NURSES.

AT Bow Street Police Court, last week, William James Jolly was charged, on his own confession, with fraud. He had been a pauper inmate of the St. Giles' Workhouse for about four years, and during that time the nurses of the infirmary had been in the habit of sending him to the bank to cash the cheques which had been given them by the guardians in payment of salary. On the 23rd of April he was handed, us usual, four cheques of the aggregate value of £9 3s. 5d., to cash. He left the workhouse, but was not seen again until he surrendered himself to the police on Tuesday. He then stated that he had spent the money travelling about, and now wished to give himself up, "because he imagined that everybody who saw him wanted him." The magistrate sent him to prison for two months with hard labour. He has certainly not got more than he deserved, and in future the nurses of St. Giles' Workhouse Infirmary will probably prefer to cash their cheques themselves, unless the guardians solve the difficulty by adopting the far more sensible plan of paying them in money.

### NORTH-EASTERN HOSPITAL.

Important building operations are being carried out at the North-Eastern Hospital for Children, Hackney Road, which will have the effect of adding 50 new beds. The nursing staff, at present 23 strong, will of course have to be strengthened, possibly, it is suggested, doubled; and it is greatly to be hoped that an additional scheme, plans for which are being prepared, will be carried into effect with as little delay as possible, since this includes a nurses' home. As things are, twelve nurses have to sleep at a house outside, in the Hackney Road, and as the house is used by the night as well as day nurses, a certain amount of discomfort is inevitable. "A nurse," to quote one of the sisters, "likes to be able to go to her own room when she is off duty."

### DISTRICT NURSES FOR GORTON.

An unfortunate misunderstanding has arisen in Gorton, where a much-needed effort has been initiated to establish a District Nursing Association. It was wisely decided not to make a start until £100 could be raised, and the appeal put forward was being generously responded to by the inhabitants, when a report got wind that the Chorlton Board of Guardians were supplying two nurses for the Gorton district. Then, of course, the movement flagged, but we trust that the publication of the actual facts will soon put matters right. The truth is that the Chorlton Guardians have consented to subscribe £10 a year to the maintenance of each nurse the Gorton people see their way to provide. This should be a material help as well as an incentive to make a point of beginning with two nurses instead of one. The number of sick poor in Gorton is already considerable, and as the population in this part of Manchester is on the increase, the start should not be delayed any longer.

### CHOOSING BETWEEN THE SISTER AND THE HORSE.

An Army Nursing Reserve sister was out riding alone, at 11 a.m., in the Northern Transvaal, close to a small town which has been held for the past year by the English. Two men on horseback, dressed in what seemed to her the uniform of our scouts, rode by. When they had gone a little farther they dismounted and, taking their rifles in their hands, came up, grasped the reins of her horse, and asked her if "she would like a walk." Surprised at the question she inquired to what regiment they belonged. Their answer made her aware that, though claiming to be British, they were really Boers in disguise. The men spoke to each other in Dutch, and then one said to her, "We are Boers and we want your horse." The sister, greatly distressed at the thought of losing the animal, which was borrowed, begged the men not to take it. They replied that they were very short of horses and required it badly. Expostulations proved unavailing; she had to give way with as good grace as possible, made easy to her by their extremely polite manner and the gentle way in which she was lifted from her saddle. They left her with the promise that the saddle and bridle should be returned. A few days after a letter was brought by a Boer to a blockhouse to say that if the English commandant would send out beyond the British lines, the sister's horse and property would be restored. He did so, and everything was found as stated, together with a letter explaining that the two men had been compelled to take the horse as they thought that the sister suspected their nationality and they feared she would ride at once to the nearest blockhouse and report their whereabouts. They had therefore, in order to secure their own safety, to choose between taking her with them beyond the blockhouses or the confiscation of her horse, and they chose the latter as being probably less repugnant to sister's feelings. No doubt the good treatment their own women have received at the hands of the British authorities tends to account for their friendly and courteous behaviour, but the sister is none the less to be congratulated upon the result of an experience that might have been most unpleasant.

### HOMES FOR LUPUS PATIENTS.

The authorities of the London Hospital receive as many lupus patients as possible, but there is not room for anything like the number requiring treatment, and homes for sufferers from the disease who cannot remain in the lupus ward appear to be a real necessity. One home of this class has now been in existence for just a year in the Mile End Road, under the auspices of Mrs. Freeman, who started with the idea of accommodating middle-class people. She has found out, however, that they prefer not to go into a home, and her four patients, three women and a boy, are all poor and paid for by friends. One is a factory girl, who is terribly disfigured and has lost the use of an eye. Mrs. Freeman, who is an energetic lady, and does all the shopping and a part of the waiting on the patients herself, encourages them to occupy themselves between the visits to the hospital—the boy by doing odds and ends of work about the home, and the women by sewing—but her experience is that the disease tends to indolence. Although Mile End Road may not be the most attractive locality for a home of any kind, proximity to the London Hospital is a great advantage in the case of lupus patients.

### SHORT ITEMS.

Princess Henry of Battenberg will open, at 54 Mount Street, on Friday next week, an exhibition of rare embroideries and miniatures, in aid of the Women's Memorial to Queen Victoria. The exhibition, which will be open for ten days, will include a sale of drawings by first-rate professional and amateur artists. — The Incorporated Society of Trained Masseuses will hold an examination on Monday and Tuesday, June 30th and July 1st, at the Trained Nurses' Club, 12 Buckingham Street, Strand. Particulars can be obtained of the secretary. No application can be received after June 12th.

### Lectures to Nurses on Anatomy.

By W. JOHNSON SMITH, F.R.C.S., Principal Medical Officer, Seamen's Hospital, Greenwich.

LECTURE XVIII.—THE BLOOD VESSELS (*Continued*).

IN the systemic circulation the blood that has been purified during its circulation in the lungs is driven by the left ventricle into a branching and widely-extended set of elastic tubes which are named *arteries*, and by these tubes is distributed to all the organs and the different structures of the body. From the smallest and ultimate arterial branches it passes into a very close network of extremely minute and almost structureless tubes called *capillaries*, and after it has traversed this network it returns to the right side of the heart through small branches to large branches, and finally through the main trunks of a second set of tubes called *veins*. It thus begins its course at the aorta and ends at either the superior or the inferior vena cava.

The distinction between *arteries* and *veins* is based not so much on their function as on their structure. The arteries as a rule have thicker, stronger, and more elastic coats than the veins, and when divided their cut ends gape whilst those of the veins collapse. Veins differ from arteries in being supplied with internal folds or valves which prevent reflux of blood (fig. 44).

The blood in the arteries is of a bright red colour, and its current is a rapid and strong one, so that when a large

FIG. 44.—Valves of veins.

arterial vessel is cut or severed the bleeding is active and profuse, and the blood is forcibly discharged in jets.

In the veins the blood is no longer bright red, but very dark red or purple. Its current, except in the main trunks, is feeble and sluggish, and when a vein is wounded, the flow of blood is continuous, and not so rapid and alarming as that from an open artery.

*The Arteries* (fig. 43).—The main trunk of the arteries is the aorta which, starting from the left ventricle of the heart, takes a horseshoe-shaped curve backwards and to the left side, forming *the arch of the aorta* (fig. 43, Lecture xvii.), then descends along the left side of the spine as far as the fourth lumbar vertebra where it divides into two large branches (*common iliac arteries*) through which arterial blood passes to the organs of the pelvis and to the lower limbs. In its downward course this large trunk passes through a special opening in the diaphragm. The straight portion of the vessel above this opening is called the *thoracic aorta*, that below the opening the *abdominal aorta*. This vessel, especially at its arch and its thoracic portion, is the frequent seat of the disease known as *internal aneurism*, which consists of a large pulsating or throbbing tumour due to weakening and consequent yielding of the arterial walls.

The aorta having recognised the obligation of the arterial system to the heart by sending two branches (coronary arteries) to this organ, gives off from its arch three large branches which transmit blood to the head and neck and to the upper limbs. The first of these is a short but very thick artery called the *innominate*, dividing into the *right carotid*, which passes upwards along the neck towards the skull, and the *right subclavian*, which curves outwards and downwards, and supplies with blood the upper limb. The *carotid* and *subclavian* arteries on the left side have no common trunk corresponding to the innominate artery, but spring directly from the arch of the aorta.

The thoracic aorta sends off to the right and left the intercostal arteries, each of which passes forwards to the front of the body along the lower margin of each rib.

When the aorta has passed through the aortic opening in the diaphragm and reached the abdomen it sends off numerous large branches to the important organs contained in this cavity. The chief of these are the *gastric*, *hepatic*, and *splenic*, all springing from a short common trunk called the *cœliac axis*, to the stomach, liver, and spleen; two *mesenteric arteries*—the superior and inferior—to the long intestinal canal; and the *renal* to the kidneys. The aorta gives off also branches which supply blood to the walls of the abdominal cavity.

These, which are but the larger and more important offshoots of the aorta, divide and subdivide, sending off both named and unnamed branches, and ultimately break up into innumerable minute vessels which gradually lose the structural characters of arteries and merge into the capillaries.

Before leaving the arteries let us follow the course of the large vessels which pass to the head and the limbs.

The carotid on each side divides just below the lower jaw into two branches: one, the *external carotid*, supplying parts outside the cranium, and the *internal carotid*, which enters the skull and supplies blood to the brain and the ear and eye.

The subclavian is continued under the name of *brachial* from the armpit to the elbow, below which it divides into large branches, the *ulnar and the radial*, both placed in front of the forearm.

Passing now to the termination of the aorta we find that the short common iliac arteries divide into two large vessels, one the *internal iliac*, which supplies the pelvic organs, the womb and its appendages, the bladder, and the terminal part of the intestinal canal; the other the *external iliac*, which runs to the front of the thigh and there becomes the *femoral*. This artery, before it reaches the knee, courses round the inner side of the limb, takes a short and oblique course in the ham under the name of *popliteal*, and divides into two arteries for the leg, the *anterior and posterior tibials* which, as their titles imply, are not both placed in front of the limb like the arteries of the forearm, but are so disposed that one supplies the anterior or extensor part, and the other the posterior or flexor part of the extremity.

The arteries of the forearm—the radial and ulnar—are joined together below the wrist by two arterial arches called the *palmar arches*. The more superficial of these arches is often accidentally wounded, and may then become the source of free and troublesome bleeding.

A similar arrangement is found in the foot where the anterior and posterior tibials form what is called the *plantar arch*.

*The Veins.*—The arrangement of the veins differs in some respects from that of the arteries. The former are more numerous than the latter and are divided into two sets, one of *superficial* or *cutaneous veins*, the other of *deep veins*. The *superficial* veins of the leg and forearm become very conspicuous in old people, and in some regions, particularly in front of the elbow, may be distinguished without diffi-

### LECTURES TO NURSES ON ANATOMY.—*Continued.*

culty in most persons. For this reason one of these vessels was formerly selected for the minor operation of bleeding and for transfusion, and is frequently used now for injections of saline solution. The deep veins are in close contact with the arteries, so that it is difficult in dissecting small arterial branches to avoid wounding the accompanying venous branches. Large arteries, such as the carotid, the subclavian, and the femoral, are accompanied by one vein, whilst the smaller arteries, such as the radial and ulnar, have each two veins. In most instances the accompanying vein or veins take the same name as the artery, but the large deep vein on each side of the neck is called *internal jugular* and not carotid. Whilst there is one large trunk—the aorta—for the arteries, the veins converge to two trunks, each of which has a separate opening into the right auricle. These large venous trunks are the *superior* and the *inferior venæ cavæ*; the former collects venous blood from all structure above, the latter from all structure below the diaphragm.

*The Pulse.*—As a healthy artery is not a rigid, but an elastic tube, it is distended and lengthened as it receives the successive waves of blood that are forced into it by the contraction or *systole* of the left ventricle of the heart. This arterial movement or pulsation as it is called may be observed in a large artery exposed in the course of a dissecting operation for the removal of a tumour, or in the flaps of an amputation wound after the blood-vessels have been tied. It can also be readily felt under the intact skin, but only in certain parts of the body where the artery lies near the surface and rests on bone. The part at which the beating or pulsation of an artery can be most easily and conveniently felt is the lower part of the front of the forearm at a spot from one to two inches above the wrist, and in a vertical line drawn upwards from the middle of the root of the fore finger. Here with a little practice one can feel distinctly the beating of the radial artery, which we all know as the pulse.

As the pulsations in the arteries keep time with the movements of the heart, the frequency of the radial beats—*the pulse rate*—corresponds with that of the cardiac beats. Consequently we would expect to find under healthy conditions a much more rapid pulse in the infant than in a subject of middle or advanced age, and, in disease, in some cases increased, in others diminished frequency. Unusual rapidity or slowness of the pulse, however, are not the only indications it affords of disease. Its force or strength may be modified so that we meet with a bounding, a hard or soft, a weak, or an almost imperceptible pulse. Again we hear of disturbances of rhythm expressed by the terms intermittent and irregular pulse, the former being distinguished by the repeated omission of a beat after a regular sequence of two or more beats, the latter by more or less disorder both in frequency and strength.

Although for most practical purposes much guidance can be thus given to those who are experienced in feeling the pulse, the results obtained by the finger may be regarded as coarse and incomplete when compared with those afforded under skilled management by the sphygmograph.

### Matrons in the Burgher Concentration Camps.

BY K. B. BRERETON, A.N.S.R., A MEMBER OF THE LADIES' COMMISSION.

At the time when the Ladies' Commission was visiting the Burgher Camps (from the latter half of July to the beginning of December 1901) a proper system of camp nursing was gradually being established. Its necessity was abundantly evident from the fact that in all camps measles either had been or was at its height; the great difficulty lay in securing the right kind of woman for the post of camp matron. In only a very few cases were successful matrons chosen from the Dutch women, doubtless owing, amongst other things, to their lack of training; on the other hand, to send to England for suitable trained women entailed a delay which it was difficult to meet at the time. Several loyal refugee women were brought up from the coast, but, with exceptions, did not prove satisfactory. A good camp matron is not an ordinary woman. Some methods, however, of relieving the overworked doctors had to be devised. In certain camps a system of reporting cases of sickness by corporals was formed. These burghers divided up the camp and took a daily list of sick people to the doctors. It can well be believed that this system had many drawbacks, over and above the ease with which a Dutch "vrow" would conceal any case of sickness which she preferred to treat herself. In other camps one of the more responsible women would volunteer, and under her Dutch girls would be chosen to seek out the cases of sickness and then report them to their head, who would hand in the report to the doctors. But this plan did not by any means fulfil all the requirements of the case. There was no trained nursing for those cases who could not, or did not, go into hospital, and who were not properly nursed in their homes; and when the matrons began to arrive for the camp the authorities issued directions for putting the whole question of the duties of a camp matron and her assistants on a proper footing.

### The Early Days of Camp Matrons.

The camp matron was to be provided with trained assistants, the number varying with the size of the camp, and was also to organise from among the Dutch girls in camp probationers to be taught their work, who would receive a small salary and uniform. A good matron working on these lines was an untold blessing to the doctors, enabling them to give more attention to those who were seriously sick, and saving much anxiety as to whether their orders would be carried out. The camp was divided out into sections, a certain number of tents being allotted to each probationer, who visited these tents daily before 8.30 A.M. and brought to her matron a written list of new cases of sickness. The matron, or her trained assistants, accompanied the doctors in their morning rounds, each probationer going with the matron and doctor in her own district, and then writing down any order which she had to carry out at once. When the matron had finished her rounds with the doctor, she arranged with her probationers what they were to do for the different patients, and as far as possible tried to teach them some of the simple rules of nursing and various ways of making the sick people a little more comfortable. In one camp an excellent matron expected her probationers to carry a broom with them, well knowing that in many cases it would be most useful. She also taught them how to tidy tents, turning out filthy rags and other accumulation, how to wash the sick, make poultices, etc. In the afternoon she visited some of the cases again herself, and later on received reports from her probationers of those patients she had ordered them to see again, ascertaining if they had received their medicines and other medical comforts ordered by the doctors. In the case of any death the matron saw that the people had material for reverent covering of the dead. In another camp, where there was an excellent matron, I visited with her and the doctor 80 cases in their tents in one morning. True, many were cases of measles pure and simple, but there were others of a much more serious kind, and all, of course, needed thought and care, if complications

### MATRONS IN THE BURGHER CONCENTRATION CAMPS.—*Continued.*

were to be avoided. This will give some idea of the work required in the early days of camp matrons, and the difficulties under which they worked were further increased, by the ignorance and superstition which they met with from the majority of the Boer women.

### Illustrations of Ignorance.

A few well-authenticated stories will suffice to demonstrate these. In one camp a mother had covered the chest and abdomen of her poor little child suffering from double pneumonia with a thick coat of varnish, making the fight for life nearly an impossible one; another, also suffering from double pneumonia, was being treated with half-crowns placed on her chest; another, an adult patient, suffering from jaundice, was rubbed with cabbage seeds: when these were planted and had grown the patient would be well! A poor little baby suffering from bronchitis was covered with fur, which had first been cut off a living cat and then roasted. These stories will go to prove how much the Boer women, with the best intention in the world, increased the anxiety of the doctors and nurses in their efforts for the nursing of the sick in camp. In every camp there was also an out-patient department where people well enough to walk came to see the doctors. Each doctor generally saw his out-patients after going to his hospital patients, and either before or after going round his section of the camp.

### The Advantages of the Matron's Round.

In a few camps the matrons did not go round with the doctor, and he left written directions for their guidance. The advantages resulting from the matron going round with the doctor were many. In the first place, her presence with the doctor helped her position with the people in the camp; she also saw the cases with him, and received his directions for their treatment on the spot, and having seen the patients with him she could, if he was prevented seeing the less serious cases again, give him a far better report, and in many ways save him the time he so often sorely needed for the very sick. Although the hospital accommodation and medical and nursing staff has been so much increased in practically all the camps, that most of the sick are treated in hospital; and nursing even for slight cases is no longer required on a large scale since the epidemic of measles has disappeared; the camp matron and her subordinates have still a very important work. There is so much for the former to do in teaching the Boer women to make the best of their present surroundings, the value of good ventilation—nothing can be more stuffy than a stuffy tent—and in instructing them in matters of hygiene, cooking, feeding, and clothing, a simple knowledge of which home duties may do so much to prevent sickness. She can lay the foundation for better future Boer mothers in her efforts to train the camp probationer in habits of cleanliness, tidiness, truthfulness, and the dignity of doing one's own work.

### Association of Asylum Workers.

### ANNUAL GENERAL MEETING.

THE annual meeting of the Association of Asylum Workers was held on Thursday last week at the Medical Society's House, Chandos Street. There was a large attendance, Sir James Crichton-Browne in the chair, and the proceedings opened with a motion by Dr. Greene, who proposed the re-election of Sir James Crichton-Browne as president. The other officers were then re-elected, and the adoption of the report, which showed that the membership in 1901 was 4,116, as compared with 2,868 in 1900, having been moved by the chairman, he said: "I think you will agree with me that it is a lucid document and shows a most satisfactory state of the Association. The thin sapling of a few years ago has grown to a considerable trunk. The increase in members is most satisfactory. I do not doubt that by a continuation of its present policy, there will be a still further growth. We must give the Association solidarity and coherence. There is no doubt that the journal of the association has fostered a sentiment of imperialism in asylum affairs. The money expended on it has been well bestowed. I attach great importance to literature as a refreshing beverage in the dulness of asylum life. I should feel inclined to make it obligatory that every nurse should read two standard novels every year. They would return invigorated to their work. The notion of exhibitions should be also attended to. There is plenty of scope for really good exhibitions. Happily the recreation of the workers is studied in a way that formerly was not the case. Possibly they will have their golf before long and even their motor cars. As regards the financial position of the Association, it is perfectly sound. I anticipate that by the end of next year we shall have a surplus to report, and it is always satisfactory to at any rate have a small surplus. The Home of Rest fund practically remains the same It is doing a most benevolent work and is well deserving of all support. A code of new rules has been drawn up, and I find them most judicious. There is no prospect of any Lunacy Bill being passed if there is any opposition, and I presume that the Bill promised in the King's speech will be opposed if it does not contain, or have added to it, a satisfactory pension clause. The Commissioners have no direct control over the asylums. When any expenses are recommended it must be remembered that an asylum on the cheap is shameful prodigality. Strict economy should be observed, but not short-sighted parsimony. The workers should be kept from the blunting effects of the asylum, and so I come back to my point about pensions. In Scotland the state of things is worse than in England. Worn out attendants are left to the mercy, if not the caprice, of the rulers of the asylum. The influence of an association numbering 4,000 is no mean thing. Individual members of Parliament, when taken alone, are almost always sympathetic, and I should advise you all to put your grievances and your dues before your representative in Parliament. I am glad to note a great change in the attitude of the press. Formerly the papers looked upon lunacy with horror. Now they look upon it as a disease. The outcome of the change on the part of the medical profession, the press and the public is that a much greater number of persons are treated under private care. There are cases that should not go to the asylum. I have no doubt that there are cases of neurasthenia where removal to an asylum might lead to life long insanity. For the poor the asylum is necessary, but for the well-to-do and affluent classes it is not. Private treatment is extending, and it can only be obtained with thoroughly trained nurses. No nurse is competent to take charge of a case without three years' asylum training, one of which should be spent in the infirmary ward. I am in favour of high remuneration, but it would be a great boon to a middle-class home if a nurse could be obtained for £1 or £1 10s. a week. I recommend the learning of massage. I have no doubt that the standard of knowledge and technical skill will continually be raised. The introduction of female nurses into male wards must help to assimilate the nursing to medical nursing. One of these days the increase of insanity and of all nervous diseases will startle the public, and then perhaps justice will be done to those who are engaged in battling with this stupendous social evil."

Dr. Hyslop seconded the adoption of the report, balance sheet, and rules, which were adopted.

*Gold Medals* were then presented to the following:—Mr. W. Hope, now inspector at Colney Hatch Asylum, 36½ years' asylum service; Miss M. Riches, head nurse at Heigham-hall, Norwich, 35½ years' asylum service.

*Silver Medals* to Mr. C. Walker, charge attendant at Caterham Asylum, 28¼ years' asylum service; Miss A. Garry, now chief nurse at Gloucester County Asylum, 28½ years' asylum service.

*Bronze Medals* were also presented to thirty others.

### Workhouse Infirmary Nursing Reform.

BY SOLOMON C. SMITH, M.D.

What one first of all has to remember is that the union is the unit of poor-law administration, and that, desirable as it may seem for some purposes, that co-operation between numbers of unions should be secured, even compulsorily, such a course would probably be a greater interference with the general routine of Poor Law administration than one could expect the Local Government Board to initiate for the rectification of nursing alone, although for other purposes such a change may come. Then, as bearing upon the same point, it must be noted that whatever may be done in large towns, where, as we know, the separate infirmary system has been found to work fairly well, it can never be feasible to remove the sick wards or infirmaries in country unions far from the wards where the aged and infirm are accommodated, because of the frequency with which the aged and infirm have, for a time, to enter these sick wards.

### Concentration of the Sick.

Any attempt to concentrate the sick from many or several unions in large infirmaries must end in either making a considerable number of the aged permanent residents in the infirmary, or in treating them during many attacks of so-called "minor ailments" in the workhouse, both of which are much to be deprecated. Moreover, putting questions of convenience on one side, I doubt whether it is wise or even humane to remove the sick, who in country workhouses are mostly aged as well as sick, far from the place in which they have been taken ill. Not only does an old pauper often have relatives near at hand, or at least within his own union, but he also often has made friends within the "house." An old labourer may break down at 60 or earlier, and, because he cannot do laborious work, he may go into the workhouse and there he may live for 10 years or more before he becomes ill enough to go into the sick wards. During those 10 years he makes friends and settles down, his character becomes known, and it seems to me quite unfair that when, at last, he becomes sick or bed-ridden, he should be shipped off to some distant "central infirmary" where he will never again have the chance of making for himself such a home as even a workhouse becomes to many a decent old pauper who lives on into old age. Therefore, I feel sure that it would be wrong to concentrate these poor old people in large central co-operative infirmaries merely for the sake of providing them with nursing. Hence in my mind the problem narrows itself down to the question how best to provide proper nursing for the sick in the small and moderate-sized workhouses; for at present we may take it that the large separate infirmaries, with their resident medical officers and their lady superintendents, are fairly well provided for. Such difficulties as arise in these great separate infirmaries are largely of a personal nature, and so far as the system is concerned, are hardly worth considering at the present moment.

### The Difficulties.

Now what are the difficulties in providing efficient nursing in workhouse infirmaries as distinct from the "separate infirmaries"? Let me run over a few. First of all there is the unwillingness of the guardians to provide it. But that is nothing. It is a mere bit of natural conservatism. Every single instance in which good nursing is provided tends to remove this difficulty in scores of other workhouses; for guardians are greatly swayed by fashion, little as they may think it. They are constantly referring to what is done in other workhouses to excuse their own shortcomings, and the more advanced guardians are as constantly doing the same to spur on their fellow guardians to better things. I by no means despair of the guardians. A far greater difficulty is that the nurse is, so far, somewhat outside the provisions which have been so carefully laid down by the Local Government Board in regard to most other officials. The arrangements made for her lodging and feeding, and for her position in regard to the patients and to the maintenance of discipline, are in a somewhat chaotic condition; they differ in every union, and far too often have been left in the hands of the master and the matron.

### The Nurse and the Matron.

The relation of the nurse to the matron is also a constant source of difficulty. This is of a two-fold nature. Partly it arises from the objection which is felt by nurses who have gone through a prolonged course of training to being placed "under" a matron whom they regard as "an outsider" so far as nursing matters are concerned. This may perhaps be regarded by some as a merely sentimental objection, and I think that sometimes it is so. But it is none the less real as a means of deterring good nurses from entering the service. No one can doubt that the growing feeling among nurses that they are members of a profession makes them stickle somewhat for what is due to them, and, that being the case, the general impression that Poor Law nursing involves subordination to an untrained matron does distinctly deter the best nurses from entering the Poor Law service. This feeling therefore, whatever be its cause or its justification, has to be reckoned with, and this difficulty has to be met if the supply of candidates is to be increased. Partly, however, the difficulty with the matron is a very real one, for there seems but little doubt that, by interfering with the order of the work, with the provision of proper assistance, and by holding back stores which the nurses consider are required for the proper care of the sick, matrons do sometimes give good reason for the dissatisfaction felt by nurses at their present position. It is not improbable, however, that other causes are at work. There is a good deal of human nature in both matrons and nurses, and until the nurse's position, and what may roughly be called her rights, are better defined than they are at present, it is evident that everything that the nurse does, which may seem to imply the exercise of authority is apt to be taken as derogating from what all matrons specially pride themselves upon possessing—namely, complete and unquestioned supremacy over all the females in the establishment; and when we add to this that what one may call the newly-fledged nurse, tinged as she sometimes is with all the pride of a newly obtained certificate, is often a younger woman than the matron, who is the wife of the master, and that she comes into a country place with all the cachet of superiority which attaches to one who has lived in large towns, it does not require much knowledge of the world to see that there is the possible making of a pretty quarrel always at hand. It is not to be wondered at then that the matron is sometimes tempted to show her power. Then there is friction; the workhouse gets a bad name, and difficulties arise in filling up vacancies. It is thus of the first importance to eliminate the conflicting authority of the matron and the nurse.

### The Meals Question.

Another cause of complaint and I may add another mode in which, without the infraction of any law, the matron may make things uncomfortable for the nurse is the existing arrangements in regard to feeding and the cooking of meals. Strictly speaking, I believe, the nurse is entitled only to rations, and how they are to be cooked and how served is a matter which is left to the matron, and this possibly accounts for a good deal. Thus, whatever else is done, if good nurses are to be attracted and induced to stay on, they ought to be enabled to feel that their duties are defined, that they are responsible directly to some other authority than the matron, and that they are in safe hands about the cooking and the serving as well as in regard to the mere quantity of their food. As to the nurses' quarters, I think that a steady improvement is on the whole taking place. As a general rule the responsibility for the quarters provided for the nurses rests with the guardians, who are doing a good deal in the matter, and where they show themselves recalcitrant I think that the Local Government Board might sometimes be induced to interfere with advantage.

(*To be continued.*)

### Native Indian Midwives.

BY A BOMBAY CORRESPONDENT.

The indigenous midwives of Bombay are drawn chiefly from the Navi or Barber caste, and from the most ignorant of the poorest Mussalmans. Their occupation is usually hereditary. The same family will send for a certain dhai or her successor for generations. When a dhai is growing old or is past work she usually takes a younger woman, of an age over 40, to the houses of her patients, to learn her methods and assist in her work. The rate of pay varies. The poorest give from 1.8 rs. to 3 rs., others 3 rs. to 5 rs., and the better class from 10 rs. to 12 rs. In addition presents of rice, pan supari, cocoanut, and sometimes a saree and cholee are given. Every class and every race employ these dhais.

### The Lack of Training.

It is the general opinion of those consulted that in Bombay there is no possible means of inducing the dhais to attend a class for instruction. Further, no doctor with a due regard for asepsis would permit them to enter the labour ward, and the character of the women, with rare exceptions, does not lend itself to training in any degree. The primary essentials in the training of a midwife, such as washing the hands with soap and water and washing the patient; not interfering unnecessarily, nor using blind force so as to endanger life; preventing hæmorrhage; giving the patient rest, fresh air, and wholesome food, are contrary to the whole practice and belief of the dhai. The dhai who consents to receive and to practise the improved methods will not be employed by those who wish for the old native methods, but by those only who would as readily employ a better trained woman.

### The Methods.

The following is the method of dealing with ordinary and difficult labour cases, and after-treatment:—As soon as a pregnant woman complains of pain, whether false or true, the dhai at once ties a string tightly round the waist of the patient above the fundus of the uterus and urges the patient to bear down. To hasten delivery the dhai sits on the bed (or floor if the patient is poor and without a bed) at the head of the patient and with hands or feet pushes with great force on the abdomen, making constant examinations per vag. with unwashed hands. A lighted sigree is placed under the bed, every window and door closed, and a cloth tied round the patient's head to prevent any air reaching her. A small seed like sago, called halun, is boiled with joggree and given as a drink, huldi (saffron) being introduced into the vagina. Immediately after delivery the dhai in some cases hastens the expulsion of the placenta by sitting on the patient's abdomen and springing up and down with great force; or the patient is made to stand up against a wall and the dhai makes rushes, and with her head butts the abdomen of the patient with force. In one case an English lady doctor, sent for to attend a Ranee, was unable to prevent this treatment. Hæmorrhage is immaterial; it is considered a good sign, and thought to keep off fever. Perhaps the most common method is for an unwashed hand to be introduced into the uterus, and with some traction on the cord the placenta pulled out. Often a piece of joggree is introduced into the os, if a portion of the placenta is retained. The cord is then cut, either by a knife or a slip of bamboo, some distance from the umbilicus, and hung in a loop round the the infant's neck. Common parcel string is used as a ligature. If the child does not cry or show signs of life, the placenta is placed on the sigree before division of the cord, and a noise is made, bell rung, etc., to rouse it. If the method employed in an ordinary case does not effect delivery, the dhai proceeds to bring down some limb, and generally succeeds in converting a vertex into a transverse position. When the patient is exhausted the friends generally call in medical aid, or convey her to hospital, usually after labour has been going on from two to five days and septic fever has begun. The dhai may terminate the case by bringing the fœtus away piecemeal.

### Duties to Mother and Child.

The patient is attended by the dhai for 10 days after delivery by a daily visit. Her duties to the mother are rubbing the body all over with sweet oil, followed by a hot bath; a lighted sigree is placed under the cot and anise seed (dry) scattered on the fire. The patient sits over it; this process is called "dhurni," and is supposed to assist lactation. A douche is considered dangerous and is never permitted. For nourishment, no milk is given, usually only soojee cooked with a great deal of ghee (butter) and joggree (coarse sugar) called siru. This "siru" is made so thick that it is eaten with the fingers. Also dry anise and other seeds, which are nibbled. The patient may walk, sit, or lie down at will from the first day of delivery. The child is placed lengthwise on the knees and rubbed thoroughly over with sweet oil; this is especially worked into the face, the features being moulded into position, the nose if small is drawn out, eyebrows raised, eyelashes curled, etc. After the oiling process, hot water is poured over, and dressing is completed by a strip of calico bound tightly round, the limbs being quite straight below.

### Appointments.

Bradford Eye and Ear Hospital.—Miss Caroline L. Jenkins has been appointed matron. She was trained at Bradford Royal Infirmary, where she has since been night superintendent and head sister.

Bushey Heath Cottage Hospital.—Miss Mabel Mary Lowe has been appointed charge nurse. She was trained at the Seamen's Hospital, Royal Albert Docks, and the London Temperance Hospital, and has since been assistant nurse at the Devonshire Hospital, Buxton, and staff nurse at the London Temperance Hospital. She has also done private nursing.

Devonshire Hospital, Buxton.—Mrs. Spalding has been appointed matron. She was trained at Crumpsall Workhouse Infirmary, Manchester, and has since held the posts of superintendent nurse at Holborn Infirmary, Mitcham, night sister at St. John's Infirmary, Hampstead, and superintendent of nurses at Exeter Workhouse Hospital.

Falkirk Cottage Hospital.—Miss H. E. Glendinning has been appointed matron. She was trained at the Royal Infirmary, Edinburgh. She has since been sister and deputy matron at Aberdeen Fever Hospital.

Lindville Asylum, Cork.—Miss S. Coffey has been appointed matron. She was trained at Sir Patrick Dun's Hospital, Dublin, where she remained on the private staff for eight years. She was for nearly one year charge nurse at the Park Hospital, London, and subsequently for three years superintendent nurse at the Leavesden Asylum, Herts. She possesses the medico-psychological certificate for mental nursing.

Royal Devon and Exeter Hospital.—Miss Rachael Cox-Davies has been appointed matron. She was trained for three years at Monmouthshire Infirmary, and has since been night superintendent, and sister of "Faith" Ward at St. Bartholomew's Hospital, London.

St. Barnabas Homes, East Grinstead.—Miss Alice Green has been appointed matron. She was trained in the Nightingale Home, St. Thomas's Hospital, afterwards being charge nurse at the York County and Wolverhampton General Hospitals. She has also done private nursing at Oxford and in London, and district nursing at Bath. She has been temporary matron at the Cottage Hospital, Caterham Valley, and since 1897 has been matron at the Sanatorium, Bradfield College, Berkshire.

Whitchurch Cottage Hospital.—Miss Emily Middlemist has been appointed matron. She was trained for three years at Guest Hospital, Dudley, and has since been sister of Children's Ward at Wolverhampton General Hospital, night superintendent at Swansea General Hospital, and night superintendent at York County Hospital.

### Action by a Nurse against a Newspaper.

DAMAGES £600.

The case of Mansell *v.* the "Sol" Syndicate, Limited and Another, was heard in the King's Bench division before Mr. Justice Grantham and a special jury on Tuesday The plaintiff, Miss Rosalie Mansell, is a certificated nurse, and the defendants are the proprietors of the *Sun* newspaper. The defendants pleaded that the alleged libel, which was contained in a report of a meeting of the Lambeth Guardians, was a fair and accurate report.

Mr. Lush, K.C., who appeared with Mr. J. R. Atkin for the plaintiff, stated that Miss Mansell was a certificated nurse, who had nursed at various institutions as well as privately, and was appointed superintendent nurse at the Renfrew Road Workhouse, Lambeth, in January, 1901. An inmate named Emily Osborne made a statement of a serious character with reference to the plaintiff, and on the agenda paper for the meeting of the guardians on July 3rd this statement was, amongst other things, set down for discussion. It was with reference to the report of that meeting that the action was brought. The Libel Law Amendment Act, 1888, for the first time gave newspapers protection as regards reports of public meetings, but the report must be fair and accurate. There was not the slightest foundation for any suggestion against the plaintiff's character, but Osborne's statement was to the effect that the plaintiff was intemperate and had admitted to her that she had had children. When the plaintiff heard of this statement she invited inquiry, and wrote letters to the clerk and to the board of guardians denying the allegations made against her. The meeting of the guardians took place, and these letters were read at the meeting immediately after Osborne's statement. Osborne had been dismissed from being the plaintiff's attendant on suspicion of taking the plaintiff's food, but she denied the charge. Mr. Lush then read the report, which was headed "Scandal at Lambeth Workhouse. Charge against a Superintendent Nurse." It gave the purport of Osborne's statement, and added that Osborne at the meeting confirmed her statement in the most specific manner. The report ended without stating whether the guardians did anything in the matter.

The Judge remarked that as he read the report it stated as a fact that the plaintiff made certain admissions, and it did not give this as a report of what Osborne said.

Mr. Lush said that the report omitted the plaintiff's denial and the statement of the medical officer that he had never seen any trace of intemperance in Miss Mansell, and it did not give the resolution of the guardians that they should proceed to the next business.

The plaintiff having given evidence in support of her counsel's opening, she was cross-examined by Mr. Germaine, K.C. She declared that her denial was not believed by the majority of the guardians. They said that they had come to no decision.

The Judge observed that that was no proof that they did not believe the denial. It was incredible, if the guardians believed the statement, that they could have allowed the plaintiff to remain.

Miss Mansell, continuing, said that as her character was not cleared she resigned.

The Judge said that if the guardians had believed the charge they ought to have referred the matter to the Local Government Board, but a resolution to that effect was negatived.

Mr. Germaine, for the defendants, contended that the report was fair and accurate, and that if an addition had been made to the report in a later issue it would have done the plaintiff far more harm. When the editor was informed of the various decisions come to by the guardians after the report left he concluded that to complete the report would do more harm to the plaintiff than to leave the balance unpublished.

Mr. Boyle, managing director of the "Sol" Syndicate, identified the flimsy sent to them by the London News Agency. The report had been sub-edited, some parts being cut out. It had been toned down. It was published in the last edition that day. On the next day they received a further report from the same agency. They thought it would be dangerous to print any more, so they left it as it was.

The Judge after examining the "flimsy" said that the word "serious" before "charge" was omitted. Apart from that it was the most moderate parts of the report that had been omitted.

Cross-examined, Mr. Boyle said that the report was fair up to the time when it left, although the plaintiff's denial was read immediately after the statement made against her.

After counsel had addressed the jury the judge summed up, remarking that the report omitted everything that was not spicy. The jury having considered the matter for 20 minutes, found a verdict for the plaintiff for £600.

### Novelties for Nurses.

BY OUR SHOPPING CORRESPONDENT.

CORONATION SOUVENIRS.

The Mazawattee Tea Company have prepared a most practical Coronation souvenir gift especially suitable for distribution amongst children. It is in the form of a small metal box of medalion shape, bearing a fine impression of the King's head, and containing a cake of chocolate. The children's ward and the little ones in children's hospitals are not likely to be forgotten at a time of general festivity, and there are few cases in which such a gift would not be welcome and permissible. Of course these little souvenirs will be readily obtainable where chocolate is sold. Tins of tea and chocolate of larger dimensions are also ready for presentation to adults.

### Presentations.

Bedfordshire Trained Nurses' Institute.—Miss Berryman on leaving the Bedfordshire Trained Nurses' Institute to take up her post as matron of the new isolation hospital, Clapham, Bedfordshire, was presented by the lady superintendent and nurses with a Queen Anne tea service, sugar tongs, and spoons in case; also a butter knife. The maids gave some pretty glass vases. Miss Berryman had been on the staff for the last 8½ years, and will be greatly missed by all.

### Where to Go.

Monday, June 2.—Grand Afternoon Concert at the Queen's Hall, Langham Place, under the immediate patronage of H.R.H. Princess Louise, Duchess of Argyll, on behalf of the Metropolitan Hospital, at 3 p.m.

Tuesday, June 3.—Drawing Room Meeting in aid of the building fund of the North-Eastern Hospital for Children, 19 Stratford Place, at 3.30 p.m.

Wednesday, June 4.—Annual meeting of the Factory Girls' Country Holiday Fund, Merchant Taylors' Hall, Threadneedle Street, at 3.30 p.m.

Lyric Theatre, Friday, June 13.—Special matinée in aid of the League of Mercy at 2 p.m. A strong company of artistes will give their services. Tickets 10s. 6d., 7s. 6d., 5s., and 4s.

### Everybody's Opinion.

**[Correspondence on all subjects is invited, but we cannot in any way be responsible for the opinions expressed by our correspondents. No communication can be entertained if the name and address of the correspondent are not given as a guarantee of good faith, but not necessarily for publication. All correspondents should write on one side of the paper only.]**

### CORONER AND NURSES.

"SISTER ELLEN" writes: The experience of a Bootle nurse being summoned to attend a coroner's court is not confined to that town alone, for in many parts of Lancashire the same injustice exists. I know of many instances where nurses have been most peremptorily "warned" to attend, without the courtesy of a subpœna being sent, and usually the unpleasant duty falls upon the night nurse who has been present at the death of the person upon whom the inquest is being held. I have frequently seen an officer calling at the hospital and requesting the nurse to be at the court within half an hour of receiving the notice, causing much inconvenience if she is on duty, and should she be on night duty she has to be awakened when she has retired for her well-earned rest and sleep; if the case is protracted or many on the list, she may have to wait until late in the afternoon, and neither is refreshment offered nor fee allowed, as she is not considered a witness. To many nurses the ordeal of appearing before a jury and taking the oath is a great hardship and quite unnerves them at their first appearance. I believe that there is no redress unless the matter can be exposed by the Press.

### THE QUESTION OF STANDING.

"MARTHA" writes: Will you kindly allow me to add a few lines on the subject of "Standing"? As a nurse I am sure there is no question that many women suffer permanently, not only from "varicose veins," but also from various forms of internal weakness due to the strain of being for so many hours obliged to stand. Of course, a nurse must expect to be a great deal on her feet, but the point I wish to emphasise is that the nurse's personal health and comfort should be considered by the hospital authorities, and that consequently it should not be contrary to "rules," written or understood, for a nurse to sit down, in either "ward" or "ward kitchen," to do any little piece of work that she can do equally well, or better, in that position! Frequently, during the afternoon, when there is absolutely no work to be done, the nurses are still required to "stand." Another much-needed reform in the same direction is to lengthen the time allowed the nurses for their "dinner." Twenty minutes is too short a time, either for digestion or rest, and yet, when two meals have to be served up within an hour, that is all that can be given. A "Medical Man" suggests that "the Staff" should complain to the "medical authorities." But that is exactly where the difficulty lies.

"A. G." writes: "K. T." puts a very pertinent question when asking "who is to fight this fight for less standing for our nurses?" and he or she who will take up arms in so good a cause will be worthy of the sincerest gratitude of all of those who have the welfare of nurses at heart. I hear it said, as a mere commonplace, "most nurses are worn out at 35," and I wonder if the people who make this remark have ever asked themselves the reason why so unnatural a state of things obtains. There is absolutely nothing inherently harmful in hospital life; on the contrary, the regular hours, plain and wholesome food, and interesting work should combine to make it essentially beneficial and most certainly would do so were it not for the over severe work required from nurses, and most of all the compulsory standing for too long periods. "K. T." is right in the remark that matrons and doctors are not told, in many cases of troubles from which nurses are suffering and for the reason given, that they believe it will hinder their chances of promotion, and they are very apt to endure evils arising from overwork and too long standing, until the constitution is affected more or less seriously and in many cases a breakdown is the result. Why, I ask again is the health of work-girls, factory hands, shop-girls (for whom it is compulsory upon shopkeepers to provide seats) so sedulously considered, while no care is bestowed upon a class so deserving as hospital nurses? If an over-tried shop-girl makes a mistake not much harm can result. A nurse has charge of human life, and we may be quite sure that conscientious as the majority of nurses are, there has been many a mistake made by them, which has had serious results, from sheer exhaustion. The fault usually rests with the council, for if they gave directions to the matron and provided her with sufficient means to avoid overworking her nurses this evil would come immediately to an end. Of course many hospitals show a bright example of consideration. In at least one great London hospital comfortable chairs are provided for the nurses, who are encouraged to rest in them whenever possible; in another, nurses are forbidden to sit down at all when on duty, and any person who is acquainted with the female interior must be well aware of the suffering inevitably attendant on so cruel a rule. The most amazing part of the whole business to my mind is the apathy of medical men on this point. They do know of these sufferings and yet make no effort to prevent them.

### MALE NURSES IN GENERAL HOSPITALS AND PRIVATE WORK.

"W. GUTTERIDGE," 23 York Place, W., writes: May I be allowed to make a few remarks in reply to "Thistle's" letter in your issue of May 10th? The trained male nurse is not by any means hoping to take the place of his female competitor; he has already done so, and unless I am very much mistaken he will continue to take her place in private work more every year. The number of female nurses who can manage a male patient physically and mentally ill is not many, and it is quite common for the doctor to send for a male nurse to take over the case: apart from this the male nurse is preferred in many cases on account of his superior power in lifting and moving the patient about. I quite agree with "Thistle" that no praise is too great for Dr. Macphail for what he has done, and continues to do, towards making the nurses as perfect as possible, but if "Thistle" is one of the "old staff," "Thistle" has failed to grasp the most important part of the work done there. The Borough Asylum may not claim to be a training school for general nursing, but if the nurse leaves the place without the knowledge of general nursing, it is the nurse's fault, and not the fault of the institution or of Dr. Macphail. I also am one of the "old staff"; for the last seven years I have been constantly employed in private work, and I have yet to see the case which I did not see at Derby in some form or other. I will go further and say that with the exception of surgical work, there is more general nursing in the asylums than in the general hospitals. "Thistle" admits that the nurses are trained to nurse skilfully any case of sickness, which means that they are well trained for general nursing. By speaking of cases likely to come under their care, "Thistle" must surely have forgotten the male and female infirmary wards, where at least thirty patients in each are requiring constant nursing. Dr. Macphail impressed on the nurses in my time the importance of improving the bodily health, and I feel sure it is the same at the present time. The patient has a better chance of recovering mentally when the bodily health is improved; hence the importance of careful nursing in the infirmary wards, and it seems to me this part should be placed first, and not second, according to "Thistle's" version.

### To Nurses.

**WE invite contributions from any of our readers, and shall be glad to pay for "Notes on News from the Nursing World," or for articles describing nursing experiences, or dealing with any nursing question from an original point of view. The minimum payment for contributions is 5s., but we welcome interesting contributions of a column, or a page, in length. It may be added that notices of appointments, entertainments, presentations, and deaths are not paid for, but that we are always glad to receive them. All rejected manuscripts are returned in due course, and all payments for manuscripts used are made as early as possible after the beginning of each quarter**

### A Book and its Story.

### THE LIFE OF A NOBLE WOMAN.*

Take joy home,
And make a place in thy great heart for her,
And give her time to grow and cherish her!
Then will she come and often sing to thee
When thou art working in the furrows, aye,
Or weeding in the sacred hour of dawn.
It is a comely fashion to be glad—
Joy is the grace we say to God.—*Jean Ingelow.*

The above beautiful lines preface the memoir of Felicia Skene, a woman of rare gifts. Endowed with unusual personal attractions, a lovely voice, mental capacity above the average, and a sympathetic large-heartedness which were invaluable to her when working in the furrows of misery and moral degradation to which those for whom she voluntarily gave the work of the best years of her life had sunk. In her own great heart joy was the irradiating force which shone through her good deeds and cheering words, bringing hope into the shadowed corners of the "silent world," as she named the prisons where she herself moved as an angel of light. Felicia Skene came of gentle antecedents. Her parents were each members of ancient Scotch families. Her mother was sister to Sir William Forbes, seventh baronet, who married Scott's first love—the lovely Williamina Stuart, to whom he had been so deeply attached in early youth. From her mother Felicia Skene inherited that charm of manner and air of distinction and high breeding, coupled with a natural gift of sympathy, which throughout her life acted as a talisman to all who came under its influence. Her quick wit and lively conversation made her very popular in society, and her extraordinary adaptability made her at home, we are told, with equal ease in the presence of a costermonger or a duke. Her father's family traced back to the eleventh century. He, James Skene of Rubislaw, was a man of considerable attainments. A travelled, cultivated gentleman, for whom the study of geology, antiquities, art, science, literature, had equal attractions. He was not only a learned man, "he was what is far better and rarer, a wise one, full of practical ability." But the great attraction of his character lay "in his gentle manner, his love of all that was beautiful in art and nature, his enjoyment of manly exercise, and, above all, in his loyal and affectionate nature."

If, as we believe, that heredity counts in the development of personality, then Felicia Skene owed much to hereditary influences, and from them she derived the unusual mental activity and breadth of mind, with the sweet reasonableness, which made her so loyal, and withal so discerning a friend to the cause or the individual in whom she was interested. Born at Aix, in Provence, in May 1821, she lived until October 1899, and from her childhood until the day of her death it may be truly said of her that she was never weary in well doing. The following extract from a recollection, written by Mr. C. W. Wood, editor of the *Argosy*, is included in the Memoir:—"Those about her gradually married, she herself would not. Probably few men were worthy of her, and she did wisely to remain single. I have every reason to think that the subject which troubles most young women—love and matrimony, and settling in life—never crossed her thoughts for an instant. . . . Suitors she could not fail to have, but all were rejected. Her life was a dream of high-souled happiness." He speaks of the power of her wonderful voice, into which, in singing, she threw all her intelligence and feeling, and which charmed her hearers as long as she possessed it. Another gift she had not always identical with a singing voice, that of a very musical one in speaking. Miss Skene's girlhood and early youth were spent in Athens. Her sister married into a Greek family of distinction, and later her parents settled for a time in Greece. "She grew to love with all the strength of her impassioned nature the Greek islands and blue waters of the Mediterranean that washed their base. She loved Athens especially, where all doors were open to her, from the palace downwards. Her spirit of adventure she kept to the very end of her life. . . . Here, too, her love of poetry was fostered, for she was deeply poetical. It seemed as if no gift had been denied her, and if actual genius was not hers, her versatility approached being nearly to it. Her gift of language was very conspicuous. Greek she spoke as a native, French also."

In 1849 an outbreak of cholera, in Oxford, taxed the limited resources of the medical authorities to the utmost. Quite recently Felicia Skene had come with her parents to reside permanently in Oxford. "The nursing of the sick in shelters was carried out under the direction of Felicia's friend, Miss Hughes (sister Marion), of whose noble efforts, for the comfort and relief of her patients, whether in body, mind, or spirit, Sir Henry Acland speaks with enthusiasm. . . The other department of the nursing, that in which Felicia took a leading part, had to be carried on in the homes of the sick, and was probably the harder and more trying of the two, from the dreadful condition of some of the yards and alleys where the sufferers lived. . . . Her quick sympathy gave her insight into the patients' needs. Her tenderness invited confidence, so that they could pour out the anxieties that weighed on their minds and retarded their recovery. Her courage and cheerfulness braced them to make an effort to get well. Her strong will ensured obedience to her orders. Her very presence, with its vigorous vitality, seemed to inspire them with hope and strength." In consequence of the devotion and capacity which Miss Skene displayed, she was made responsible for engaging and dismissing a band of women, by the order of the deputy-chairman of the Board of Guardians, who were to be instructed in their duties as nurses. In 1854 came a second outbreak of cholera, accompanied by small-pox. Then Miss Skene's invaluable services were again called into requisition, and she entered with the same self-effacing devotion into the service of the sick and dying as in the former outbreak. This was the time of the Crimean war, and finding how successfully her band of women had worked at home in the cholera cases, she conceived the idea of sending them out to Scutari. She became Miss Nightingale's agent in Oxford, and it was her wish to go out in charge of the nurses. But such an idea would not be entertained by her family, to whom, in the early fifties, such an undertaking was out of the question for a young and gently-nurtured woman. Of Felicia Skene's work among the prisoners and the fallen, space does not permit mention. To this very interesting memoir of a very remarkable woman we must refer our readers, and we feel sure that they will echo the words of the author (E. C. Rickards) "that it is a happiness to have been brought as it were into her presence by the study of her noble life." Miss Skene was a frequent writer in The Hospital on prison and other social topics.

* "Felicia Skene: a Memoir." By E. C. Rickards. (Publisher: John Murray, London. Illustrated. 1 vol.)

### Wants and Workers.

The District Nurse, Whitstable, thanks the two kind donors of parcels of old linen for use among poor patients.

Would any nurse like to have The Hospital forwarded on to her, the same day as published? If so, please send a post card to Miss Hilda T. Towle, 76 Tideswell Road, Eastbourne.

District Nurse, Maple Cottage, Earl's Colne, Essex, would be grateful to anyone who could tell her where to obtain a secondhand pair of spring crutches.

### Echoes from the Outside World.

### The Coronation.

ONE of the most interesting of the royal guests for the Coronation arrived at Southampton on Saturday afternoon in the *Dunottar Castle* in the person of King Lewanika, the paramount chief of the Barotse kingdom. He is a very black gentleman, tall, well built, about 50 years of age, with an extremely intelligent face. He comes of a long line who for centuries have ruled Barotseland. He assumed power at the end of 1877, but in 1884 a revolt took place, and he had to fly for his life. He remained a year in exile, and came into power again in 1885, after a series of desperate conflicts. Since 1890 his kingdom has been practically, and in 1897 definitely, under British protection, Lewanika now receiving an annual subsidy from the Chartered Company. The sable sovereign, who wore a light flannel suit and a white felt hat, on landing was handed by a representative of the Colonial Office a letter of welcome from Mr. Chamberlain on behalf of the King, who expressed a hope to see him soon after his arrival in London. This letter appeared to afford King Lewanika great pleasure, and he said that he was glad to be here as paramount chief of the Barotse kingdom, but "more glad that he should see the King personally." On Tuesday the paramount chief visited London in order to be measured for his Court costume, but he is staying in Somersetshire for the present.

### Foreign.

THE Queen of Holland has made such excellent progress that she is now able to leave her bed twice a day, and rest on a couch for three-quarters of an hour. She takes sufficient nourishment, and, according to the official bulletins, the desire to do more brain work grows in proportion to the increase in her strength. The Queen will shortly proceed to Germany, and will make a lengthened stay in the neighbourhood of Ems.

The death of Lord Pauncefote, the British Ambassador at Washington, which took place on Saturday morning, deprives the diplomatic service of a distinguished member. He was a native of Gloucestershire, and started his career in the legal profession, becoming Attorney-General of Hong Kong. In July, 1874, he was appointed Chief Justice of the Leeward Islands, and was knighted in the same year. As a diplomatist he was discovered by Mr. Disraeli, who in September of the same year brought him home and installed him as Under-Secretary of State for the Colonies, transferring him two years later to the Foreign Office. Here he soon made the mark that his chief anticipated, and in 1882 he became Permanent Under-Secretary of State for Foreign Affairs, in succession to Lord Tenterden. In 1889 he was appointed to represent England at Washington, and remained there ever since, receiving a peerage in 1899 in recognition of his eminent services. In appearance he was more like a country squire than an ambassador, and his manner was brusque rather than polished. But he was a great diplomatist and held in universal esteem. On Wednesday there was a State funeral, and the remains of the deceased were taken under military escort to Rock Creek Cemetery and deposited in a temporary vault. Subsequently, at the instance of President Roosevelt, a national ship will bring the body home to England. Lord Pauncefote leaves a widow and four daughters, but no heir.

### Art.

THE death of M. Benjamin Constant occurred in Paris on Monday afternoon. The celebrated French painter was only in his fifty-sixth year, and though he had been ailing for some time past, his life was not supposed to be in any immediate danger. He had been an exhibitor at the Salon since 1869, where he sent in a scene from "Hamlet," but of late years he had devoted his energies almost entirely to portrait painting. Two of his canvases, one of Lord Savile and the other of M. De Blowitz, are on the walls of the Salon this year, but he is best known to Englishwomen because of his remarkable portrait of the late Queen Victoria, which, after appearing in the Salon in 1900, was by command of the King hung in the place of honour at the Royal Academy in 1901. The picture, it may be remembered, represented the Queen as seated alone in the House of Lords, holding the insignia of power, and attracted much attention. Quite recently M. Constant received a letter from Queen Alexandra in which she expressed the hope that his health would soon permit him to come to England, adding that she would wait until he did so to visit the Grafton Gallery, so that he might do the honours in regard to his own pictures.

### Sanitation.

The awards to successful candidates at the examination of the National Health Society were given on Saturday afternoon by the Princess Christian at Grosvenor House. Lord Derby in his speech alluded to the striking evidence of the power which the society had gained, as shown by the fact that, during the past year, its students had once more met with considerable success in securing public appointments as sanitary inspectors, health visitors, and the like. The ladies who had obtained these appointments had placed themselves in positions to carry on the work of the society, and there were many cases in which the knowledge could be better imparted by ladies than by the surgeon, doctor, or casual visitor. Sir William Church said that in the last 30 years he had seen extraordinary changes in the sanitary condition of the country, though he confessed that he looked with a little suspicion upon physiology taught in Board schools; and Sir Frederick Treves emphasised the superficial character of some so-called teaching by alluding to an answer at a recent examination. The candidate had been asked as to the formation of bile, and the reply was that "it formed in the stomach, and was used for cleaning carpets." It is an interesting commentary on their proceedings that the list of candidates who have successfully passed the examination of the Sanitary Inspectors' Examination Board which qualifies them to serve as inspectors under the Public Health Act, includes more than a dozen women, several being trained by the National Health Society.

### For the Children.

The King and Princess Louise, Duchess of Argyll, have consented to be the patrons of the International Bazaar for the Welfare and Protection of Children, which will be held in the Guildhall, London, in the third week in July, under the presidency of Lord Beauchamp. There will be three sections: Sir James Crichton-Browne presiding over the medical, and Lord Cross over the legislative. The third will be educational and philanthropic. The congress will be a continuation of a series inaugurated at Paris some years ago, and held in various continental cities, including Florence and Budapest. It will be attended by representatives of the colonies, the United States, and various European countries. Many boards of guardians and most of the leading organisations dealing with children in the United Kingdom will send delegates, and the President of the Local Government Board is one of the vice-presidents of the congress.

### A New Domestic Movement.

AN organisation called the "Guild of Dames of the Household" has come into existence. Its mission is to train and send out "dames" who will be proficient in the various departments of domestic service, "to lift up the work of domestic service to an honourable profession." The "dames" are to wear a uniform in the home—a blue drill gown, white linen apron, and a white "Dora" cap, with a "willow pattern" badge. The "willow pattern" is the badge of the guild. The members of the Guild are instructed by a teaching staff, aided by working mothers who lend their babies to the nursing department for an entire afternoon every week. As to off-time, "dames" ask for an hour every day in which they can do as they like, half a day a week to themselves, and every alternate Sunday.

### For Reading to the Sick.

"SHOW ME THY FACE."

Show me Thy Face!
The heaviest Cross
Will then seem light to bear;
There will be gain in every loss,
And peace with every care:
With such light feet
The years will fleet,
Life seem as brief as blest,—
Till I have laid my burden down,
And entered into Rest.

Show me Thy Face,—
And I shall be
In heart and mind renewed,
With wisdom, grace, and energy,
To work Thy work endued.
Shine clear, though pale,
Behind the veil,
Until, the veil removed,
In perfect glory I behold
The Face that I have loved.

*C. B. Macready.*

"I am the Light of the World. He that followeth Me shall not walk in darkness, but shall have the Light of Life."—John vii. 12.

There is one light which shows me the footprints of Christ, and another light which only begins to dawn upon me when I am treading in the footsteps of Christ and going after Him. The first points out to me the way of life, the second lightens for me the darkness, which overtakes even the faithful traveller.

"Whoso followeth Me shall not walk in darkness;" only the Light can say this of itself. Encouragement enough to follow Him, and consolation enough for those who follow Him.

He who goes before me, and promises to light His follower on the way with a light that never goes out, carries—what can have no attraction for the carnal man—the Cross before me, and invites me also to carry the Cross after Him.

To carry the Cross after Christ in the days of health means nothing else than to die to sin.

To carry the Cross after Christ in the days of sickness means especially to follow in submission and patience Him our example of submission and patience.

And if I carry this Cross after Him, if I follow Him in submission and patience, then a new light will arise for me upon the way of the Cross, and chase away the darkness which proceeds from my sick body, from the uncertainty of the future, and from the gloomy chambers of death, and bring into my heart the blessed morning of glad confidence and cheerful expectation.

If Christ is always with His own, the Christian will have even on his sick bed a Friend who will not flee when death knocks, but, leaving him only dust for a prey, will lead the spirit, freed from the dust, into the eternal Home with Him.

This faith, *Christ with me always*, sweetens many a bitter hour for me now, and will sweeten for me the bitterest of all, when I shall cry: "Lord, abide with us, for it is toward evening."—*Johann M. Sailer.*

Yes, like a ray of sunlight,
His Word shines through the gloom,
And after winter's darkness
Comes spring in fresher bloom;
And after vainly searching,
We find a resting meet;
For rest, and hope, and glory
Are found at Jesus' feet.—*Anon.*

### Notes and Queries.

**The Editor is always willing to answer in this column, without any fee, all reasonable questions, as soon as possible.**
**But the following rules must be carefully observed:—**

1. **Every communication must be accompanied by the name and address of the writer.**
2. **The question must always bear upon nursing, directly or indirectly.**

**If an answer is required by letter a fee of half-a-crown must be enclosed with the note containing the inquiry, and we cannot undertake to forward letters addressed to correspondents making inquiries. It is therefore requested that our readers will not enclose either a stamp or a stamped envelope.**

*Stains.*

(69) Would you kindly tell me if it is possible to get the stains of ergot out of bed-linen, and also the stains of permanganate of potassium from marble slabs?—*Nurse Lawrence.*

Ask your local chemist. Or try salts of lemon for the ergot stains, but be very careful in using it, as it is a strong poison. Wash the marble with Sapolio and then put chloride of lime mixed to a paste on the stain, leave it until the next day; repeat the process until the stain disappears.

*Weir-Mitchell.*

(70) Can you tell me if there are any homes for the Weir-Mitchell treatment in the South of England, or in Derbyshire? If so, will you kindly give the names of the places?—*L. H.*

There are numerous homes all over the country where this treatment may be obtained, but as we never recommend private institutions you will have to seek what you want by advertisement.

*Home.*

(71) Can you tell me of a home or institution where a gentleman of education, between 70 and 80 years of age, whose antecedents make the idea of the parish workhouse repugnant to him, could be admitted? He suffers from senility and has no control over his functions. His friends would be glad to contribute 15s. weekly for his board.—*G. W. M.*

See the list of homes for Chronic and Incurable cases in "Burdett's Hospitals and Charities." Possibly some cottage nurse might undertake the case privately.

I am anxious to place a girl of eleven, subject to epileptic fits in a home. Only a nominal sum could be paid.—*Meta.*

The Meath Home of Comfort for Epileptics, Westbrook, Godalming, receives suitable girls from 8s. a week.

Can you kindly recommend a home, where a man 33 years of age suffering from locomotor ataxia, could be received permanently, free? Also will you tell me if there is any way of seeing a copy of "Burdett's Hospitals and Charities" without buying it?—*M. M.*

He might or might not be eligible for one of the charities for Chronic and Incurable cases. Write to the Secretaries of the Incurable Hospitals at Putney or Streatham. 2. You might see it at the office of the publishers, The Scientific Press.

*Pharmacy.*

(72) Will you kindly tell me which are the head colleges in London at which nurses can learn pharmacy and dispensing?—*M. G. F.*

Apply to the Secretary of the Pharmaceutical Society, 17 Bloomsbury Square, S.W.

*Cottage Nursing.*

(73) Will you kindly tell me to whom I ought to apply about cottage nursing, and also say upon what principles the system is worked.—*Sister.*

Apply to the Affiliated Benefit Nursing Associations, 26A Buckingham Palace Road, S.W.

*Hospital Training.*

(74) I have had several years' experience in fever nursing, and am now anxious to take up maternity nursing. Will you kindly tell me if I ought to be trained in general nursing first, and if so, for how long do you advise me to train?—*B. F.*

A certificate for general nursing is always an advantage, but with your experience in nursing a year's training in general work and the usual course of midwifery instructions ought to be enough for your purpose.

**Standard Nursing Manuals.**

"The Nursing Profession: How and Where to Train." 2s. net; post free 2s. 4d.
"Art of Massage." (Second Edition.) 6s.
"Elementary Physiology for Nurses." 2s.
"Elementary Anatomy and Surgery for Nurses." 2s. 6d.
"Practical Handbook of Midwifery." 6s.
"Surgical Ward Work and Nursing." Revised Edition. 3s. 6d. net; post free 3s. 10d.
"Mental Nursing." 1s.
"Art of Feeding the Invalid." 1s. 6d.

All these are published by the Scientific Press, Ltd, and may be obtained through any bookseller or direct from the publisher, 28 and 29 Southampton Street, London, W.C.

### Travel Notes.

**By Our Travelling Correspondent.**

### C.—WHAT WE CAN DO FOR FIVE POUNDS.

### Caen.

There are many of us whose monetary resources for a holiday are very limited, and, alas! whose time devoted to idleness is but very short in the twelvemonth. I purpose, therefore, to give you a series of articles on Continental places which you may visit for a week on the small sum of £5, which shall cover travelling expenses as well.

Sometimes perhaps it will be necessary to curtail the seven days to five to keep within the stipulated sum, but then too many of us have only the week out of which must come two days for the journey.

First of all, then, let us take Caen for a beginning. The journey is not expensive, second-class return with saloon on steamer, £1 5s. By arrangement there are, I think two or three hotels that would take you for 6fr. or 7fr. per day. Seven francs per day is, roughly speaking, £2 per week, that with the journey will make only £3 5s., leaving you £1 15s. for tips and excursions, and you will do the week in comfort.

### How to Spend the Week.

You now go direct by steamer viâ Newhaven, so there is no time lost by railway travelling, and I should advise your devoting your mornings to the churches and architectural beauties of Caen, and your afternoons to excursions. Apart from the magnificence of the ecclesiastical buildings there are numberless picturesque houses in the old streets, forming delightful subjects for sketches or for the kodak. The town has lost much of its picturesque appearance by the covering up of canals and diversion of the river; a few years since, as one can see from pictures, the beautiful church of St. Pierre at the east end rose abruptly from the water and the effect was grand and beautiful, now, alas! a modern Boulevard skirts that end of the building.

### William's and Matilda's Expiatory Offering.

As they had married within the prohibited degrees, the conqueror and his bride sought to appease Heaven in the person of an offended Pope by building the magnificent twin churches of the "Abbaye aux Hommes" and "Abbaye aux Dames." William began his Church of St. Etienne in 1064, and the work was overlooked by Lanfranc. Before the high altar is a small and insignificant slab which marks the ruthless conqueror's tomb, although it has been so often rifled that nothing of the poor skeleton now remains there. Probably the story of his death and burial is known to most, but its incidents are so dramatic as to tempt me to relate them. By his cruel orders a sack of the city of Mantes was ordered, which he superintended. His horse stepping on some of the red-hot embers stumbled, and William was thrown with violence on the peak of the saddle, such as soldiers used in those days. He was taken to the Priory of St. Gervais in Rouen, where he died in great agony, and deserted by all, even his sons leaving the dead body uncared for. An unknown knight with Christian charity brought the dead man to St. Etienne, and a tomb was prepared before the high altar, but even now he was not to be buried in peace. To build the church he had seized and appropriated without payment the land of one Asselin, who at the funeral denounced the oppressor and demanded the price of the ground. The Bishop, horror struck, hastily paid the money and the service proceeded. The bearers, however, probably unnerved by these sinister interruptions, allowed the coffin to strike against the side of the vault as it was lowered, when, to the terror and dismay of all present, the wood burst and the body of the mighty soldier, exposed to view and far advanced in decomposition, was to all senses a thing so horrible that, without further religious ceremony, the unhonoured remains were hastily covered from sight. So died and was buried the powerful conqueror who had ruled by cruelty and oppression, and whose idea even of love-making was comprised in the simple method of dragging Matilda about by the hair and thumping her head against the wall when she would not promise to be his wife—at least so say the chroniclers, but we may hope there is some exaggeration in this tale.

### The Abbaye aux Dames.

To visit the choir and crypt it is necessary to go first to the hospital of the Hôtel Dieu, because the nuns using this part of the Abbey it is closed to the general public, except by entrance through the hospital, one of the oldest in the world, probably founded by our Henry I. Cecilia, daughter of William and Matilda, was the second Abbess. At the consecration of the church her parents made an offering of her to the church, and placed her upon the altar in token thereof. We do not hear much of Matilda except with regard to her wonderful work in the Bayeux tapestry, of which I hope to speak another day. Probably her life was not very happy with her turbulent lord, though the aforesaid chroniclers affirm that he really loved Matilda with all the strength of which he was capable; perhaps she knew of noble traits and kind acts which have not been handed down to us, for surely in almost all natures there is the divine spark, and it is best discerned by those who love us best.

Her grave, like that of her husband, has not been undisturbed. In 1562 the Huguenots desecrated it with wanton brutality, and in 1793 what remained was swept away by the Revolutionists, when the marble slab marking the spot was the only thing left.

### Charlotte Corday and Beau Brummel.

At 148 Rue St. Jean once lived Charlotte Corday, though born elsewhere. In this house she matured her plans for the taking off of the monster Marat, and however much we may deprecate the method of his death, human nature must rejoice in the end of such a monster. The Girondins used to meet in the Rue des Carmes, and probably it was there that Charlotte was made to contemplate the possibility of her deed.

Beau Brummel was once Consul at Caen, and he died there in great poverty and misery, and was buried in the Protestant cemetery. I think he outlived George IV., so we may hope that the sin of leaving his former friend to die in wretchedness cannot be laid to his charge.

Next week I hope to finish the account of Caen, and to say something of Bayeux, the latter place easily visited from Caen.

### TRAVEL NOTES AND QUERIES.

Schwalbach and other Places for Anæmia (Nurse Mary).—First of all let me impress upon you the absolute necessity of taking a doctor's advice as to a suitable place. It is true that Schwalbach and other places are good for anæmia, but there are probably other reasons which would make a great difference in the selection, so you must not choose for yourself. There is, however, no harm in hearing of different spas and explaining to your doctor which would suit your purse best. There is no chance of your getting work of any sort to do, nor would the doctors allow you to do it, part of the cure consisting in resting. Your board and lodging might by arrangement be had for 5s. 6d. per day. The course of baths will cost, roughly speaking, £2. Other small charges at the springs, etc., another 10s. Doctors' fees are not high, and very probably, with their usual generosity, they would charge you nothing. There are several other places suitable, La Bourboule, Franzensbad, St. Moritz, Bagnères de Bigorre, etc.; but Schwalbach is the most reasonable and the easiest to reach.

Paris for one Night (J. B.).—Go to the Hôtel Britannique, 20 Avenue Victoria; ask for room on third floor. It is a little difficult to advise you how to spend your time, because I don't know your tastes. First you must see Notre Dame; this will take you (to see superficially) one hour, and the Madeleine should be visited also; for the other churches I fear you will have no time. Well, then the Louvre cannot be passed over, though you cannot spare more than two hours or so. Then the "Invalides," where Napoleon's tomb is. That, I think, is about all you will have time for; but at night hire an open fiacre and drive round Paris. Take the man by the hour, and tell him to drive you round the busiest and gayest streets. If you have time to see the Gobelin Tapestry Factory do so: it is close to the Jardin des Plantes. You will find "Paris, and How to See It," a shilling book of Messrs. Henry Gaze and Sons, all you will need as a guide-book.

Antwerp for a Fortnight (A. M. A.).—I think you would find a fortnight rather long unless you are sketching. It is not a good centre for excursions. Why not stay a week at Antwerp and a week on the Meuse—say at Dinant—for the other week? No, you cannot manage for yourselves for such a short time, and unless you speak French well it would not answer. Write for rooms on third or fourth floor to the Hôtel du Commerce, 10 Rue de la Bourse, or Hôtel d'Or, 1 Ruelle des Moines. Terms from 7 francs per day (5s. 10d.). I should advise four clear days there, and the rest either at Brussels or in the Ardennes, Dinant, or La Roche.

## Figures and Tables

**Fig. 44. f1:**